# Pollution Characteristics and Assessment of Carcinogenic and Non-Carcinogenic Risks of Volatile Halogenated Hydrocarbons in a Medium-Sized City of the Sichuan Basin, Southwest China

**DOI:** 10.3390/toxics14050370

**Published:** 2026-04-25

**Authors:** Xia Wan, Xiaoxin Fu, Zhou Zhang, Yao Rao, Mei Yang, Jianping Wang, Xinming Wang

**Affiliations:** 1 School of Environment and Resource, Southwest University of Science and Technology, Mianyang 621010, China; wanxia0056@126.com; 2 Sichuan Foreign Environmental Cooperation Center, Chengdu 610031, China; raoyao1700@126.com (Y.R.); yangmei0287@126.com (M.Y.); wangjianping957@126.com (J.W.); 3 Changsha Center for Mineral Resources Exploration, Guangzhou Institute of Geochemistry, Chinese Academy of Sciences, Changsha 410013, China; 4 State Key Laboratory of Advanced Environmental Technology, Guangzhou Institute of Geochemistry, Chinese Academy of Sciences, Guangzhou 510640, China; wangxm@gig.ac.cn

**Keywords:** volatile halogenated hydrocarbons, ozone-depleting substances, short-lived halogenated hydrocarbons, source apportionment, health risk assessment, the Sichuan Basin

## Abstract

Volatile halogenated hydrocarbons (VHHs) are critical air toxic pollutants, with some ozone-depleting substances (ODSs) strictly regulated by the Montreal Protocol. However, current understanding of the pollution characteristics, sources, and health risks of atmospheric VHHs in Southwest China remains insufficient. This study performed field observations of atmospheric VHHs in summer in Mianyang, a medium-sized industrial city in the Sichuan Basin. Freon-12 (563 ± 20 ppt) and Freon-11 (264 ± 15 ppt) were the most abundant chlorofluorocarbons (CFCs); chloromethane (785 ± 261 ppt) and methylene chloride (563 ± 505 ppt) dominated among VSLSs. The mean concentration of regulated ODSs (1037 ± 33 pptv) was notably lower than unregulated very short-lived chlorinated substances (1887 ± 745 pptv), reflecting effective ODSs phase-out locally, yet enhancements relative to Northern Hemisphere background implied potential leakage from residual tanks. Methylene chloride and trichloroethylene concentrations exceeded global background levels by over 10 times, indicating strong anthropogenic industrial influences. Phased-out CFCs displayed negligible diurnal variation due to stringent emission controls, whereas unregulated VSLSs exhibited a distinct U-shaped diurnal cycle, with peaks driven by morning boundary layer dynamics and evening accumulation. Positive matrix factorization revealed that industrial sources, including electronic solvents (28.6%), industrial processes (27.8%), and solvent usage (23.7%), accounted for 80.1% of total VHHs. The total carcinogenic risk (2.3 × 10^−5^) surpassed the acceptable threshold (1 × 10^−6^), dominated by 1,2-dichloroethane, chloroform, carbon tetrachloride, and 1,2-dichloropropane. All individual compounds exhibited mean hazard quotients (HQs) below the non-carcinogenic risk threshold. The cumulative hazard index reached 1.5, suggesting combined non-carcinogenic risks to the local population. These results support VHHs health risk management and ODSs control in Southwest Chinese industrial cities.

## 1. Introduction

Volatile halogenated hydrocarbons (VHHs) refer to hydrocarbons containing one or more halogen substituents in place of hydrogen atoms, such as Freon-11, Freon-12, chloroform, and tetrachloroethylene. These compounds are widely employed in modern industrial processes. Notably, chlorofluorocarbons (CFCs) and very short-lived substances (VSLSs) play pivotal roles within this group and have significant implications for stratospheric ozone depletion, the greenhouse effect, and human health [1,2,3]. Representative CFCs include Freon-11, Freon-12, and Freon-113, all of which are colorless, odorless liquids or gases that were once widely used as refrigerants, blowing agents, and industrial solvents. CFCs are distinguished by their chemical inertness and long atmospheric lifetimes, which enable their transport to the stratosphere, where photochemically released halogens catalyze ozone destruction [4]. This process leads to long-term adverse effects on both ecosystems and human health [5]. In response, the international community formally adopted the Montreal Protocol on Substances that Deplete the Ozone Layer in 1987, establishing a phased reduction schedule for ozone-depleting substances (ODSs). ODSs are compounds that contain chlorine (Cl) or bromine (Br) atoms and possess atmospheric lifetimes sufficiently long to allow transport to the stratosphere. ODSs undergo ultraviolet photolysis to release chlorine (Cl•) or bromine (Br•) radicals, which catalytically destroy stratospheric ozone. CFCs serve as a representative class of ODSs [6]. Besides their ozone-depleting potential, CFCs are potent greenhouse gases, with 100-year global warming potentials (GWP_100_) ranging from several times to tens of thousands of times that of CO_2_ [7]; consequently, they are subject to regulation under the Kyoto Protocol to the United Nations Framework Convention on Climate Change [8]. VSLSs are halogenated gases whose atmospheric lifetimes are shorter than six months, such as chloromethane, methylene chloride, and chloroform. Although VSLSs are not regulated by the Montreal Protocol due to their short atmospheric lifetimes, recent studies indicate they significantly contribute to ozone depletion in the lower stratosphere [9]. Toxicologically, inhalation exposure to ambient CFCs and VSLSs has been linked to neurological and immunological impairments, as well as carcinogenic risks following chronic exposure [10,11]. Notably, among VSLSs, trichloroethylene and methylene chloride have been classified by the International Agency for Research on Cancer (IARC) as Group 1 and Group 2B carcinogens, respectively.

To systematically assess the effectiveness of ODSs control measures, many countries have established continuous monitoring programs and developed global background observation networks. Notable examples are the Earth System Research Laboratory (ESRL) network operated by the National Oceanic and Atmospheric Administration (NOAA) and the Advanced Global Atmospheric Gases Experiment (AGAGE) network led by the National Aeronautics and Space Administration (NASA). Long-term observational data reveal a sustained decline in global atmospheric CFCs concentrations [7]. Since acceding to the Montreal Protocol in 1991 and completing the phase-out of CFCs by 2010, China has implemented extensive monitoring of volatile halogenated hydrocarbons. These efforts have yielded substantial environmental benefits, including the avoidance of approximately 5.8 million ODP-tonnes of Freon-11-equivalent emissions and 23 billion CO_2_-equivalent tonnes of greenhouse gases, with atmospheric CFCs showing a pronounced decreasing trend [12,13,14]. Regional studies have corroborated these trends. For instance, Zeng et al. (2020) reported that CFCs decline rates in the Pearl River Delta (PRD) exceeded those at the Mauna Loa (MLO) background site between 2001 and 2018, with sources linked to refrigeration and CFCs substitutes [15]. Yi et al. (2021) observed a decline in Freon-11 from 302 pptv (in 2004) to 254 pptv (in 2011) across five major cities [16]. Research in Beijing indicated that only Freon-11 and Freon-12 exceeded background levels, primarily due to regional transport and leakage from CFCs banks [17]. More recently, Huang et al. (2024) reported significant declines in the atmospheric mixing ratios of Freon-11, Freon-12, Freon-113, and Freon-114 in Guangzhou between 2010 and 2023, with estimated rates of −4.7, −4.3, −0.9, and −0.02 pptv a^−1^, respectively [18]. Furthermore, during 2022–2023, the mixing ratios of these phased-out compounds remained within 1–5% of Northern Hemisphere background levels [18]. Notwithstanding these overall reductions, concerning anomalies have emerged. Recent studies have identified a slowdown in the decline rate of Freon-11 in eastern and northern China, as well as unexpectedly high mixing ratios of controlled substances such as carbon tetrachloride and bromomethane, attributed to inadvertent emissions, biomass burning, and refrigerant production [19,20,21,22]. Furthermore, emissions of certain VSLSs, notably methylene chloride and chloroform, are increasing [23,24]. Elevated concentrations of chloromethane and tetrachloroethylene were detected at the Mount Tai background site, while high levels of methylene chloride were reported in Hangzhou [25,26]. Therefore, intensive monitoring of regional concentration levels, growth characteristics, and source apportionment of both phased-out CFCs and rapidly increasing VSLSs is essential for refining ODSs compliance assessments and strengthening pollution control of these toxic and hazardous substances. Currently, observational studies on atmospheric CFCs, VSLSs, and other volatile halogenated hydrocarbons in China have predominantly concentrated in major urban agglomerations such as the Yangtze River Delta, Pearl River Delta, and Beijing-Tianjin-Hebei region. In contrast, research in Southwest China remains limited.

The Sichuan Basin, a key economic zone in Southwest China, encompasses major cities such as Chengdu, Chongqing, and Mianyang. This region features a complex economic structure and rapid modernization, leading to extensive historical usage of ODSs across sectors such as refrigeration, building insulation foam, electronics manufacturing, and agriculture. These legacy CFCs may continue to be released from existing equipment and products, exerting an ongoing impact on atmospheric composition. The region’s basin topography, combined with its stagnant, humid climate, creates unfavorable conditions for pollutant dispersion, leading to the accumulation of hazardous chlorinated halocarbons with potential adverse health effects. A comprehensive assessment of volatile halogenated hydrocarbons in non-megacities of the Sichuan Basin is urgently needed. This study aims to (1) elucidate the characteristics of controlled ODSs and non-controlled VSLSs based on field samples collected on consecutive hot summer days (Section 3.1); (2) analyze the pollution levels by comparison with background values (Section 3.2); (3) identify the main sources of VHHs using a positive matrix factorization (PMF) receptor model (Section 3.3); (4) evaluate the carcinogenic and non-carcinogenic risks posed by toxic VHHs via Monte Carlo simulation coupled with sensitivity analysis (Section 3.4). The results could provide essential data to support informed ODS compliance strategies and environmental management in the region.

## 2. Materials and Methods

### 2.1. Field Sampling

**Figure 1:**
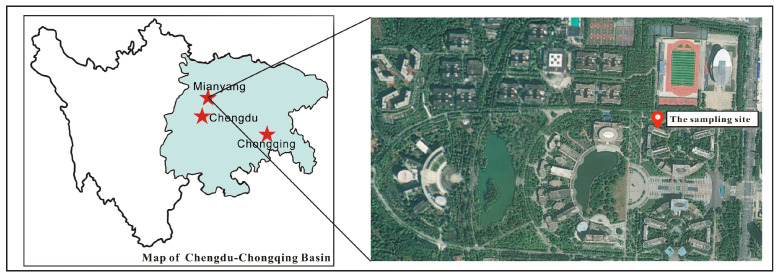
Location of the sampling site (Mianyang) in the Sichuan Basin.

The sampling campaign was conducted in Mianyang, a key industrial city in the northwestern Sichuan Basin and the second-largest economy in Sichuan Province, lying 120–300 km from the metropolitan cores of Chengdu and Chongqing. To characterize ambient levels of volatile halogenated hydrocarbons in a typical medium-sized urban environment within this basin, samples were collected at the rooftop (about 20 m a.g.l.) of the East Seventh Teaching Building (31.533° N, 104.700° E), Southwest University of Science and Technology (Figure 1). During the sampling period, the average temperature was 30.8 ± 3.0 °C and relative humidity averaged 54.2 ± 13.8%. Prevailing winds were northerly to northeasterly, with mean wind speeds of 2.1 m/s. The sampling site lies in Fucheng District, which occupies the southeastern portion of the city. Although Fucheng accounts for less than 3% of the city’s total land area, it generates 35.6% of Mianyang’s overall GDP and 45% of its industrial output [27]. The sampling site is situated within a school teaching area, surrounded mainly by teaching and administrative buildings. There are no major point sources, such as thermal power plants or large industrial facilities, within a 10 km radius. Located just two kilometers from the site is the national urban air quality monitoring station operated by the China National Environmental Monitoring Centre. Furthermore, because the sampling location lies directly downwind of Mianyang’s principal industrial zones, it could serve as a reliable indicator of regional emissions originating from industrial sources.

Ambient air samples were collected using 3 L Summa canisters(Entech Instruments Inc., Simi Valley, CA, USA) coupled with flow-controlled integral samplers operating over 1 h sampling periods. Prior to the sampling, all canisters were subjected to seven consecutive cleaning cycles using high-purity nitrogen in an automated canister cleaner, with heating applied to facilitate contaminant removal. After cleaning, canisters must be kept under a vacuum of <6 Pa until sampling. Following cleaning, five canisters were randomly selected, filled with high-purity nitrogen, and analyzed following the same analytical protocol applied to field samples; the resulting blank values for all target compounds were below the method detection limits. Flow rates of the integral samplers were calibrated prior to each sampling campaign. Annually, we verify canister airtightness and the inertness of the inner wall coating by analyzing low-concentration PAMS standards and TO-15 standards (0.1 ppbv), with the acceptance criterion being less than 10% concentration decay over a 30-day continuous analysis period.

Sampling was performed during 15–24 August 2019, excluding days with heavy precipitation (19–21 August). The summer sampling window was deliberately chosen to maximize detection of ODSs emissions associated with peak demand for refrigeration and air conditioning, while circumventing confounding factors such as the extended “West China Autumn Rainfall” season and potential emission reductions due to production halts associated with heavy pollution emergency responses during autumn/winter. On each sampling day, seven 1 h samples were collected at approximately 2 h intervals (08:00–09:00, 10:00–11:00, 12:00–13:00, 14:00–15:00, 16:00–17:00, 18:00–19:00, and 20:00–21:00). A total of 49 samples were collected. Meteorological parameters including wind direction, wind speed, temperature, and relative humidity were continuously recorded using a micro-meteorological station (Vantage Pro2, Davis Instruments Corp, Hayward, CA, USA) co-located at the sampling site.

### 2.2. Chemical Analysis

Ambient air samples were analyzed for volatile halogenated hydrocarbons following the U.S. Environmental Protection Agency (EPA) Method TO-15, employing a preconcentration system (Entech 7200, Entech Instruments Inc., Simi Valley, CA, USA) coupled with a gas chromatograph-mass spectrometer (7890/5977 GC/MS, Agilent Technologies, Santa Clara, CA, USA). Detailed analytical procedures and instrumental parameters are described in our previous paper [28]. Briefly, target compounds in the air samples were enriched through a three-stage cryogenic trap system, which effectively removed the majority of carbon dioxide and water vapor (removal efficiency > 95%). Chromatography separation was performed on a DB-1 capillary column (60 m × 0.32 mm × 1.0 μm, Agilent Technologies, Santa Clara, CA, USA) under the following temperature program: initial hold at 10 °C for 3 min; ramp to 120 °C at 5 °C/min; then to 250 °C at 10 °C/min, held for 7 min. Helium carrier gas was delivered at a constant flow of 2 mL/min, with an injector temperature of 180 °C. The MS was tuned with PFTBA prior to each analytical sequence, requiring that the relative abundances at *m*/*z* 69, 219, and 502 meet manufacturer specifications and that the full width at half maximum (FWHM) fall within 0.45–0.65 amu. Air and water background was monitored at *m*/*z* 28 and *m*/*z* 18, respectively, and an acceptable level was defined as a peak area less than 10% of the abundance at *m*/*z* 69. MS detection was performed in electron ionization (EI) mode at 70 eV. The interface temperature, ion source temperature, and quadrupole temperature were maintained at 250 °C, 230 °C, and 150 °C, respectively, with mass spectra acquired over a scan range of 20–300 amu. For statistical validity, only compounds detected in >60% of samples (14 species) were retained for subsequent analysis.

Calibration standards were prepared using a high-precision dilution system (Entech 4700, Entech Instruments Inc., Simi Valley, CA, USA). A primary TO-15 standard gas mixture (Spectra Gases Inc., Branchburg, NJ, USA) at 1 ppbv was dynamically diluted to generate five secondary calibration standards at concentration levels of 0.5 ppbv, 0.1 ppbv, 10 pptv, and 2 pptv, along with a zero-air blank. These standards were analyzed following the same analytical protocol as environmental samples to establish calibration curves. All target compounds exhibited correlation coefficients (R^2^) exceeding 0.995, confirming excellent linearity and method stability. Prior to sample analysis, system blanks were analyzed to verify the absence of contamination. Single-point calibration checks at 2 pptv were performed daily; recalibration was conducted if deviations exceeded ±10% of the initial calibration curve. Compounds identification was achieved by matching retention times and characteristic ion profiles in the NIST database, while quantification employed an external standard method with internal standard correction. A fixed-concentration internal standard mixture (bromochloromethane, 1,2-difluorobenzene, chlorobenzene-d5, and 4-bromofluorobenzene; 10 pptv) was introduced to compensate for instrumental variability. Method detection limits (MDLs) were determined according to HJ 168-2020 by performing seven replicate analyses of a low-concentration standard (2 pptv) under identical conditions; MDLs were calculated as three times the standard deviation of the replicate measurements. For the 14 target species, the analytical method exhibited precision within 5% RSD, spike recoveries ranging from 92.1% to 110.7%, and MDLs between 2 and 17 pptv [29].

### 2.3. Health Risk Assessment

Quantitative health risk assessment was performed following the protocols recommended by the U.S. Environmental Protection Agency and China’s Ministry of Ecology and Environment to evaluate potential adverse effects via inhalation exposure to volatile halogenated hydrocarbons [30,31]. Both carcinogenic and non-carcinogenic risks were characterized.

The exposure concentration (EC) for each target compound was derived as:

(1) $$\mathrm{EC}=\frac{\mathrm{CA}\;\times \;\mathrm{ET}\;\times \;\mathrm{EF}\;\times \;\mathrm{ED}}{\mathrm{AT}}$$

where CA is the ambient mass concentration of the volatile halogenated hydrocarbon (μg m^−3^), ET is the exposure time (h d^−1^), EF is the exposure frequency (d a^−1^), ED is the exposure duration (a), and AT is the average time (h). Parameters were adopted from the Exposure Factors Handbook of Chinese Population (Adults) and USEPA health risk assessment guidelines: ET = 24 h d^−1^, EF = 365 d a^−1^, ED = 74.8 a, and AT = 74.8 × 365 × 24 h.

Carcinogenic risk was expressed as the lifetime cancer risk (LCR):

(2) LCR = EC × IUR

where IUR is the inhalation unit risk (μg m^−1^)^−1^, representing the upper-bound excess lifetime cancer risk per unit concentration of the pollutant. IUR values for each compound were derived from the carcinogen classification database of the International Agency for Research on Cancer [32]. The acceptable threshold for lifetime carcinogenic risk to the general adult population is 1 × 10^−6^; risks below this level are considered negligible, whereas risks exceeding 1 × 10^−4^ indicate definite carcinogenic concern.

Non-carcinogenic risk was evaluated via the hazard quotient (HQ):

(3) $$\mathrm{HQ}\;=\;\frac{\mathrm{EC}}{\mathrm{RfC}\times 1000}$$

where RfC is the inhalation reference concentration (mg m^−3^). Reference concentrations were primarily obtained from the USEPA Integrated Risk Information System [33], supplemented by data from the California Office of Environmental Health Hazard Assessment [34] when IRIS values were unavailable.

To account for potential additive or synergistic effects of multiple pollutants on human health, the cumulative hazard index (HI) was calculated by summing the individual hazard quotients:

(4) $$\mathrm{HI}\;=\sum_{i}{\mathrm{HQ}}_{i}$$

where i denotes each compound included in the non-carcinogenic risk assessment. An HI value exceeding 1 indicates a potential non-carcinogenic health risk to the exposed population, while an HI ≤ 1 suggests negligible non-carcinogenic risk.

### 2.4. Monte Carlo Simulation and Sensitivity Analysis

Monte Carlo simulation method was employed for risk assessment to minimize the uncertainty arising from single-point measurements of various parameters [35,36]. The variable parameters included the mass concentrations of volatile halogenated hydrocarbons (CA), exposure time (ET), exposure frequency (EF), exposure duration (ED), averaging time (AT), exposure concentration (EC), inhalation unit risk (IUR) and chronic reference concentration (RFC), each of which was characterized by a probability distribution. In this study, lognormal distribution had best fit for most measured concentrations of volatile halogenated hydrocarbons based on Anderson–Darling test. The distributions for the other parameters were derived from the previous studies [36,37]. During a single trial, values were randomly selected from the defined probabilities for each uncertain variable and then the model output was calculated. To assess the convergence and the stability of the simulation results, we conducted the iteration count at 10,000 and repeated the process 100 times. Monte Carlo error (MCE) was the standard error of the average outputs from the 100 times replications. SD was the minimum standard deviation of the outputs across the 100 simulations. MCE/SD quantifies the uncertainty of the Monte Carlo simulation relative to the variability of the output’s simulated distribution, and a ratio below 5% is acceptable [38]. We found that the MCE/SD ratios for LCR and HQ were 1.63% and 2.17%, respectively, which met the requirements. R Version 4.4.1 was employed for the Monte Carlo simulations.

A sensitivity analysis was conducted to identify parameters with significant influence on the estimated carcinogenic risk and chronic toxicity risk. The analysis was implemented in R Version 4.4.1 using the sensitivity package (Version 1.30.2) [39]. The Sobol total-order index provides a comprehensive measure to identify input variables that have a significant influence on the uncertainty of the model output, either by themselves or through interactions with other variables.

### 2.5. PMF Receptor Model

The positive matrix factorization (PMF) model is a receptor modeling approach widely employed for source apportionment of ambient air pollutants [40]. The PMF model decomposes the matrix X of observational data into a source contribution matrix G, a source profile matrix F, and a residual matrix E:

(5) $$X_{ij}=\sum_{K=1}^{p}g_{ik}f_{kj}+e_{ij}$$

where *x_ij_* represents the concentration of compound *j* in sample *i*, *g_ik_* denotes the contribution of source k to sample *i*, *f_kj_* is the fraction of compound *j* in the source profile of source *k*, and *e_ij_* is the residual.

The model determines the optimal source contributions and profiles by minimizing the objective function Q:

(6) $$Q=\sum_{i=1}^{n}\sum_{j=1}^{m}\left[\frac{x_{ij}-\sum_{K=1}^{p}g_{ik}f_{kj}}{u_{ij}}\right]$$

where *u_ij_* is the uncertainty associated with the concentration of compound *j* in sample *i*.

Source apportionment was conducted using the EPA PMF 5.0 model. Data processing procedures were as follows: missing values were imputed with the median concentration of the corresponding compound; values below the method detection limit (MDL) were substituted with MDL/2, with associated uncertainties assigned as 5/6 of the MDL; for quantified concentrations above the MDL, uncertainties were calculated as:

(7) $$\sqrt{{\left(0.1\;\times \;\mathrm{Concentration}\right)}^{2}+{\left(\mathrm{MDL}\right)}^{2}}$$

## 3. Results and Discussion

### 3.1. Variation Characteristics of VHHs Mixing Ratio

**Figure 2:**
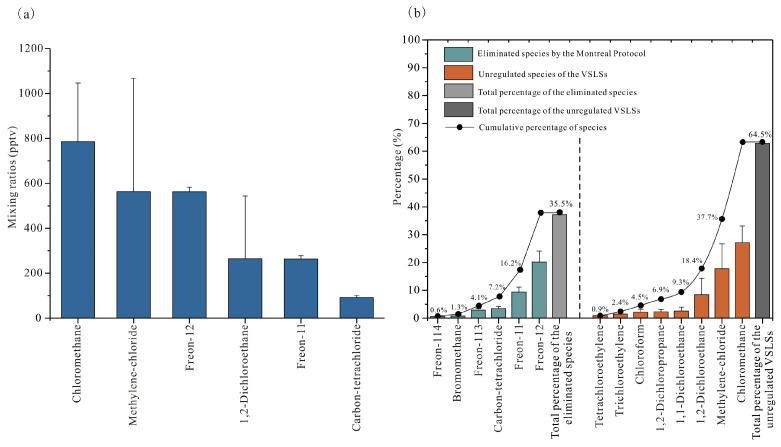
(**a**) The six most abundant volatile halocarbon species among the 14 measured species and (**b**) the percentage contributions for all 14 species during the summer of 2019.

In this study, we present an analysis of 14 VHHs in the summertime ambient air of Mianyang, a typical medium-sized city within the urban agglomeration. The target compounds included six CFCs phased out under the Montreal Protocol (Freon-12, Freon-114, bromomethane, Freon-11, Freon-113, and carbon tetrachloride), and eight uncontrolled, chlorine-containing VSLSs (chloromethane, methylene chloride, 1,1-dichloroethane, chloroform, 1,2-dichloroethane, 1,2-dichloropropane, trichloroethylene, and tetrachloroethylene). During the observation period, the mean mixing ratio of total VHHs was 2924 ± 763 pptv (mean ± SD), ranging from 1937 to 6125 pptv. Montreal Protocol-regulated CFCs averaged 1037 ± 33 pptv, accounting for 35.5% of the total VHHs, whereas non-regulated VSLSs species averaged 1887 ± 745 pptv, comprising 64.5% (Figure 2). As illustrated in Figure 2a, the six most abundant VHHs observed were chloromethane, methylene chloride, Freon-12, 1,2-dichloroethane, Freon-11, and carbon tetrachloride. Among these, the top three compounds, chloromethane, methylene chloride, and Freon-12, collectively accounted for 65.4% (1911 ± 615 pptv) of the total VHHs. A comparison of standard deviations (SD) reveals that the variability for the three dominant Montreal Protocol-regulated CFCs (Freon-12, Freon-11, carbon tetrachloride; range: 9.4–20.0 pptv) was markedly lower than that for the three major uncontrolled VSLSs species (chloromethane, methylene chloride, 1,2-dichloroethane; range: 261.1–504.8 pptv). This indicates that the concentrations of the Montreal Protocol-controlled substances remain relatively stable, a pattern consistent with their prolonged atmospheric lifetimes (>30 years) and the slow release of these controlled substances from existing equipment banks [41]. Figure 2b presents the relative contributions of individual species: among the regulated CFCs, Freon-12 (563 ± 20 pptv, 19.2%), Freon-11 (264 ± 15 pptv, 9.0%), and carbon tetrachloride (92 ± 9 pptv, 3.1%) were the most abundant; among the non-regulated VSLSs, chloromethane (785 ± 261 ppt, 26.9%), methylene chloride (563 ± 505 pptv, 19.3%), and 1,2-dichloroethane (265 ± 279 pptv, 9.1%) dominated. Collectively, these six compounds comprised 86.6% (2531 ± 701 pptv) of the total observed VHHs.

Table 1 summarizes the mean mixing ratios (pptv), atmospheric lifetimes, ozone depletion potentials (ODP), and 100-year global warming potentials (GWP_100_) for 14 target compounds, together with summertime VHHs concentrations reported from other typical urban environments for comparison. Among the phased-out substances under the Montreal Protocol, Freon-12 exhibited the highest average mixing ratio (563 ± 20 pptv) during our observation period. This concentration, while lower than the 2008 Guangzhou value (593 ± 8 pptv), was notably elevated relative to measurements obtained from major Chinese urban centers since 2012—namely, a five city composite (Beijing, Hangzhou, Chengdu: 526 ± 20 pptv), Chongqing (420 pptv), Hong Kong (508 ± 7 pptv), and the Yellow River estuary background site (529 ± 14 pptv) [15,16,21,29,42]. Meanwhile, the Freon-11 concentration (264 ± 15 pptv) fell below those measured in Guangzhou (272 ± 3 pptv), Chongqing (320 pptv), and the Yellow River estuary background site (274 ± 26 pptv), yet exceeded those observed in major metropolitan areas during 2015–2019 (244 ± 28 pptv) and Hong Kong (245 ± 5 pptv). The concentrations of Freon-12 and Freon-11 recorded in this study appear elevated when compared to those reported for other major Chinese cities in recent years. This observation is likely attributable to the local circumstances in Mianyang: the progress of obsolete equipment phase-out, temperature-driven fugitive emissions from operational legacy equipment, and differences in the enforcement of Montreal Protocol-mandated phase-out strategies at the local administrative level. The measured mixing ratio of carbon tetrachloride (92 ± 9 pptv) fell below observations from eastern Chinese sites, including the Yellow River estuary background site (117 ± 37 pptv) and the Zibo area (290 pptv), yet exceeded those recorded at Mount Tai (85 ± 7 pptv) and Hong Kong (85 ± 12 pptv) [25,42,43,44]. Notably, the mixing ratio of bromomethane in Mianyang (21 ± 1 pptv) is comparable to earlier observations in Guangzhou (2004: 21 pptv) and Chongqing (2012: 20 pptv), yet exceeds recent levels in Beijing (15 pptv), Mount Tai (12 ± 2 pptv), Hangzhou (12 pptv), and the Pearl River Delta (17 ± 0.8 pptv) [25,26,42,43,45,46]. Bromomethane has diverse sources, and several recent studies have revealed emerging emission sources along with a significant fraction of “unknown source” [47]. Thus, further work is required to identify the unknown sources responsible for the elevated concentrations in this region. The ambient level of Freon-113 (81 ± 3 pptv) in this campaign was elevated relative to the observations from Guangzhou (66 ± 3.8 pptv), Mount Tai (75 ± 4 pptv), and Hong Kong (76 ± 24 pptv) during 2015–2020. However, the mean Freon-114 concentration (17 ± 4 pptv) was comparable to those measured at regional background sites: the Yellow River estuary (17 ± 0.6 pptv), Mount Tai (17 ± 0.4 pptv), and Nam Co in Tibet (15 pptv) [21,25,43,44,48].

For unregulated VSLS species, the ambient chloromethane level recorded in this study (785 ± 261 pptv) was lower than those measured at eastern Chinese urban and regional background sites, namely the Yellow River estuary (1879 ± 1336 pptv), Mount Tai (848 ± 262 pptv), Hangzhou (912 pptv), and Beijing (800 pptv), yet exceeded the concentration reported for Nam Co (381 pptv) [21,25,26,47,49]. For methylene chloride (563 ± 504 pptv) and chloroform (64 ± 39 pptv), the observed concentrations were also lower than those reported for major metropolitan areas such as Beijing (890 pptv and 360 pptv, respectively), Hangzhou (2207 pptv and 129 pptv, respectively), and Hong Kong (821 ± 59 pptv and 43 ± 3 pptv, respectively) [26,43,50]. These relatively low concentrations of chloromethane, methylene chloride, and chloroform in Mianyang, when compared with the developed regions of eastern China, are likely attributable to the weaker industrial emission intensities in this area.

**Figure 3:**
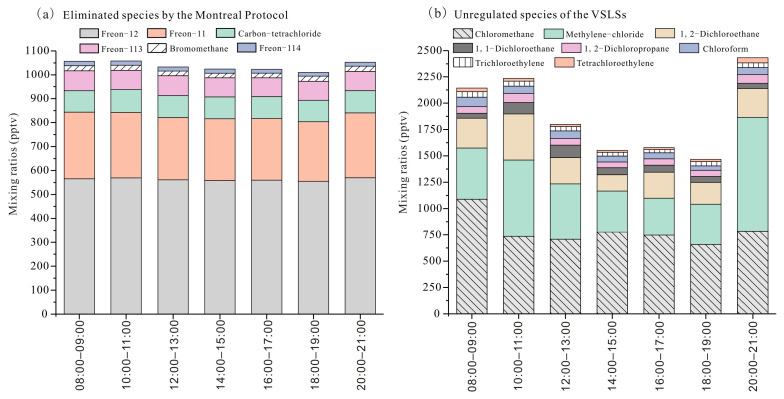
Diurnal variations of (**a**) Montreal Protocol-phased-out chlorofluorocarbons (CFCs) and (**b**) uncontrolled chlorinated very short-lived substances (VSLSs) observed in Mianyang during the sampling campaign.

Figure 3 illustrates the diurnal variations in ambient concentrations of phased-out CFCs and uncontrolled VSLSs in Mianyang during the observation period. Phased-out CFCs exhibited minimal diurnal variability (Figure 3a). However, Freon-11 exhibited the most pronounced diurnal variation, with a standard deviation of 10.6 pptv in hourly mean mixing ratios—two to 12 times higher than those of other CFCs species. This is primarily due to these substances having been completely phased out by the government for approximately 10–15 years, with emissions from the use of quotas in specific application scenarios also being strictly controlled. Historically, Freon-11 was the second most abundant CFCs in ambient air, with major sources including foam blowing (for polyurethane and polyethylene production), air conditioning refrigerants, and aerosol propellants prior to the implementation of the Montreal Protocol [41]. The distinct diurnal cycle of Freon-11 suggests ongoing emissions from the use or leakage of legacy products within the study area. For the uncontrolled VSLSs (Figure 3b), ambient concentrations typically peaked during the morning and evening hours, resulting in a distinct U-shaped diurnal pattern. The first diurnal peak was observed at approximately 11:00, consistent with the influence of a rising planetary boundary layer and intensified photochemistry, as noted by [51]. Concentrations subsequently declined throughout the afternoon, reaching a minimum between 15:00 and 18:00. As photochemical activity weakened toward evening, the ensuing decline in boundary layer height allowed unregulated halocarbons to accumulate, culminating in a second peak at 20:00. This diurnal pattern closely resembles those documented in previous studies conducted in Beijing and Zibo [43,46].

**Figure 4:**
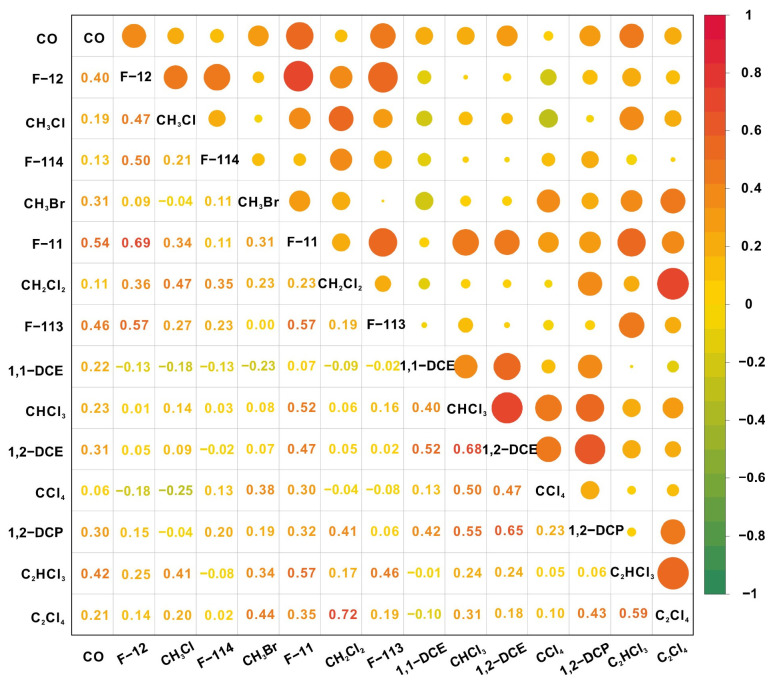
Linear correlation heatmap among 14 volatile halocarbons and the tracer CO.

Absolute concentrations of volatile halocarbons are subject to considerable variation arising from differences in meteorological conditions, emission sources, and spatiotemporal factors. Consequently, the slopes and correlation coefficients derived from linear regression against tracer species serve as reliable indicators for elucidating regional source emission characteristics [45,52]. Figure 4 presents a correlation heatmap for the 14 target VHHs and the selected tracer. During the sampling period, statistically significant correlations were observed between Freon-12 and Freon-11 (R = 0.69, *p* < 0.05), Freon-12 and Freon-113 (R = 0.57, *p* < 0.05), and Freon-12 and Freon-114 (R = 0.50, *p* < 0.05). Historically, Freon-12 and Freon-11 were widely employed as refrigerants, while together with Freon-113, they also served as spray propellants and closed-cell foam blowing agents [53]. These strong correlations suggest that these species share similar sources and analogous usage scenarios.

Among the uncontrolled VSLSs, a statistically significant linear correlation was observed between chloromethane (CH_3_Cl) and methylene chloride (CH_2_Cl_2_) (R = 0.47, *p* < 0.05), the two most abundant species in this category. Both compounds exhibited pronounced U-shaped diurnal variations, suggesting that they may be influenced by anthropogenic emissions from similar sources. Previous studies have identified chemical manufacturing, solvent use, and fossil fuel combustion as the primary anthropogenic sources of CH_3_Cl in China [54]. Carbon monoxide (CO), a chemically stable tracer primarily originating from incomplete combustion, serves as a typical indicator for emissions from incomplete combustion of fossil fuels or biomass. During the observation period, the slope of the linear regression between CH_3_Cl and CO was 1.4 ± 0.4, exceeding the emission ratios of 0.54–0.85 reported in the literature for typical source profiles of biofuel burning (e.g., grassland and agricultural residues) [55,56]. This discrepancy suggests that ambient CH_3_Cl in Mianyang during summer may be influenced by contributions from multiple pollution sources. The source profile of CH_3_Cl during the sampling period exhibited distinct weekday–weekend variations, as documented in our previous work [57]. On weekends, CH_3_Cl correlated strongly with CO (R = 0.73, *p* < 0.01), a tracer of incomplete combustion; in contrast, on weekdays, it correlated significantly with xylene (R = 0.56, *p* < 0.05), a marker of industrial solvent emissions. This divergence suggests that during the sampling campaign, CH_3_Cl was likely influenced more substantially by industrial solvent emissions on weekdays, whereas contributions from fossil fuel and biomass combustion became more prominent on weekends.

### 3.2. Comparison of Mixing Ratio with Northern Hemisphere Background Values

**Figure 5:**
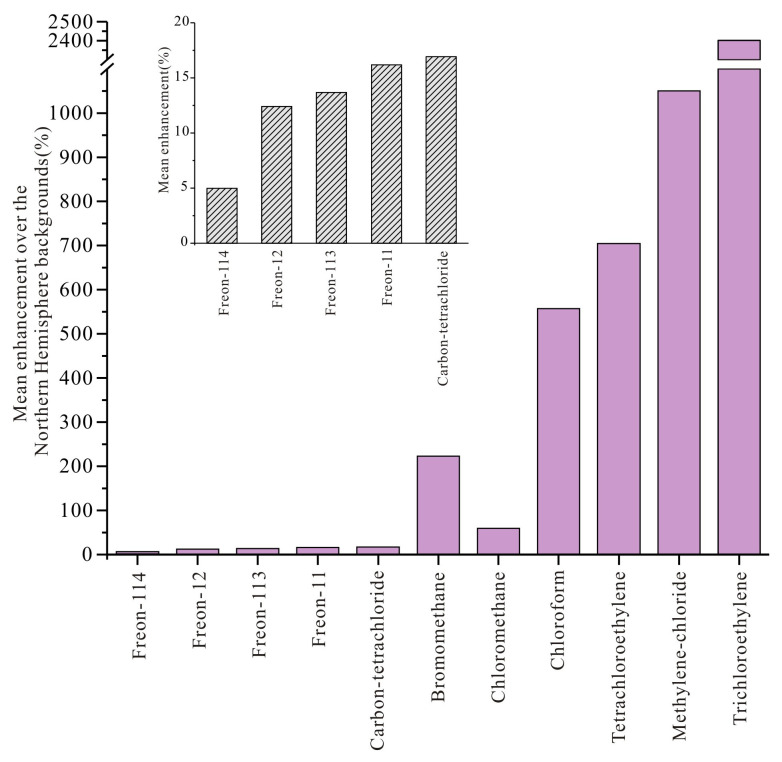
Percentage enhancements of mean mixing ratios relative to global background levels for volatile halocarbons in Mianyang.

Figure 5 presents the percentage enhancements of mean mixing ratios relative to global background levels for six phased-out CFCs substances and five uncontrolled VSLSs species in Mianyang. To ensure temporal consistency with the field campaign, global background concentrations were extracted for 17–27 August—coinciding with the sampling dates—from three monitoring stations: Mauna Loa (19.539° N, 155.578° W) of the NOAA Global Monitoring Laboratory (GML), and Jungfraujoch (46.548° N, 7.990° E) and Monte Cimone (44.193° N, 10.701° E) of the NASA Advanced Global Atmospheric Gases Experiment (AGAGE) network. For trichloroethylene and Freon-114, background values were obtained from the corresponding seasonal periods of 2007 and 2017, respectively, due to data availability constraints. Throughout the study period, all target species exhibited concentrations consistently above global background levels. Notably, the enhancement factors for Montreal Protocol phased-out CFCs species ranged from 5% to 16%, markedly lower than the 59% to 2401% for uncontrolled VSLSs, with bromomethane, also subject to Montreal Protocol-regulated substance, falling outside this range at 223%.

In accordance with relevant compliance provisions, China has frozen the production and consumption of CFCs since 1999 and has progressively phased them out thereafter. By 2010, the production and consumption of major ODSs, including Freon-114, Freon-113, Freon-12, Freon-11, and carbon tetrachloride, had been reduced to zero. Among the phased-out species examined in this study, Freon-114 exhibited the smallest enhancement relative to global background concentrations (5%, 1 pptv). The primary sources of Freon-114 were its use as a propellant and refrigerant, with the Ministry of Environmental Protection estimating its national usage at less than 0.12 kt in 1995 [58]. Consequently, after more than two decades of comprehensive phase-out, ambient Freon-114 concentrations in Mianyang were found to be comparable to global background levels. Freon-113, primarily employed as a cleaning agent for precision components, exhibited an enhancement of 13.7% (10 pptv) relative to background concentrations. Previous studies have documented a declining trend for this compound, with concentrations decreasing at an annual rate of approximately 1.2 pptv since 2000 [21,59], and national emissions in 2007 were estimated at less than 0.8 kt [60]. Thus, the modest elevation of Freon-113 in Mianyang may be partly attributable to measurement or analytical uncertainties under extremely low concentration conditions, as well as potential emissions from uncontrolled applications [14].

Atmospheric emissions and concentrations of Freon-12 and Freon-11 declined rapidly throughout the 2000–2010 period. However, several studies have reported a slowdown in the rate of decline for both their emissions and concentrations since 2012, attributing this phenomenon to “unexpected emissions” originating from eastern China [16,20,61]. In this study, Freon-12 and Freon-11 exhibited enhancements relative to global background concentrations of 12.4% (62 pptv) and 16.2% (37 pptv), respectively. The enhancement observed for Freon-12 was greater than that reported for the Yellow River estuary region in 2017 (3.7%, 19 pptv), while remaining comparable to those for Hong Kong (14.5%, 72 pptv) and Beijing (10%) [17,21,44]. For Freon-11, the enhancement measured in Mianyang was smaller than those reported for both the Yellow River estuary region (18%, 41 pptv) and Beijing (50%) [17,21]. Thus, the elevated concentrations of Freon-12 and Freon-11 relative to background levels in Mianyang may be linked to the continued release of “banked” substances, i.e., refrigerants from existing cooling appliances and blowing agents from insulating foams, which are presumed to remain sequestered in equipment currently undergoing gradual phase-out [62].

In Mianyang, ambient carbon tetrachloride exhibited an enhancement of 16.9% (13 pptv) relative to global background concentrations. Carbon tetrachloride emissions are primarily attributed to feedstock production, industrial chemical agent usage, landfills, and ongoing releases from legacy contamination [63]. Prior studies have documented a significant decline in both carbon tetrachloride emissions and atmospheric concentrations in China following the deepening of Montreal Protocol phase-out efforts after 2000 [21,61]. However, some investigations have identified a notable increase in emissions from parts of eastern China since 2012, attributed to fugitive releases during feedstock usage [64]. Comparatively, the enhancement of carbon tetrachloride above background levels observed in Mianyang (16.9%) substantially exceeds those reported for Singapore in 2011–2012 (10%), Hangzhou in 2021 (7%), and Hong Kong in 2020–2021 (11%), suggesting that carbon tetrachloride in this region may reflect either ongoing local emissions or regional transport contributions [26,43,65].

It is noteworthy that among the phased-out CFCs substances observed in this study, bromomethane exhibited the highest enhancement relative to global background values (223%, 15 pptv). As an Article 5 country, China was required to freeze anthropogenic production and consumption of bromomethane in 2002, achieve a 20% reduction by 2005, and complete full phase-out by 2015, with exemptions allowed only for quarantine and pre-shipment (QPS) applications. It is noteworthy that although non-QPS bromomethane consumption was scheduled for elimination by 2015, China obtained critical use exemptions (CUEs) for ginger cultivation during the 2015–2018 period, effectively postponing the phase-out of non-QPS use until 2019. Compared with other regions, the enhancement of bromomethane in Mianyang relative to background concentrations remained elevated (223%, 15 pptv), with corresponding values of 142% (10 pptv) for the Yellow River estuary region, 121% (8 pptv) for Hong Kong, and a range of 29–114% for Hangzhou. A recent study has revealed that bromomethane emissions in China during 2020 were 13 times higher than those of Freon-12 (expressed as equivalent concentrations), with a substantial fraction of these emissions remaining poorly constrained [47]. In Mianyang, the prevalent use of soil fumigation prior to 2019, the extensive cultivation of rapeseed, and the routine practice of biomass/biofuel burning all constitute plausible contributors to the observed bromomethane enhancements [47]. Further investigation will be necessary to enable a quantitative source attribution.

During the study period, among the uncontrolled VSLSs observed, the most pronounced enhancements relative to global background concentrations were these of trichloroethylene (2401%), methylene chloride (1050%), and tetrachloroethylene (704%). Notably, the enhancements for methylene chloride and trichloroethylene exceeded those reported for the Yellow River estuary region and Hong Kong, where values ranged from 347% to 857% [21,44]. Chlorinated solvents, predominantly released from anthropogenic activities such as industrial processes, solvent applications, and chemical production, have shown a marked upward trend in both emissions and atmospheric concentrations in recent years [23,26]. A recent study indicates that this rapid rise in atmospheric chlorinated solvents threatens to partially offset the reduction benefits achieved for ODSs and slow the recovery of the ozone layer [66].

### 3.3. Positive Matrix Factorization Source Apportionment

**Figure 6:**
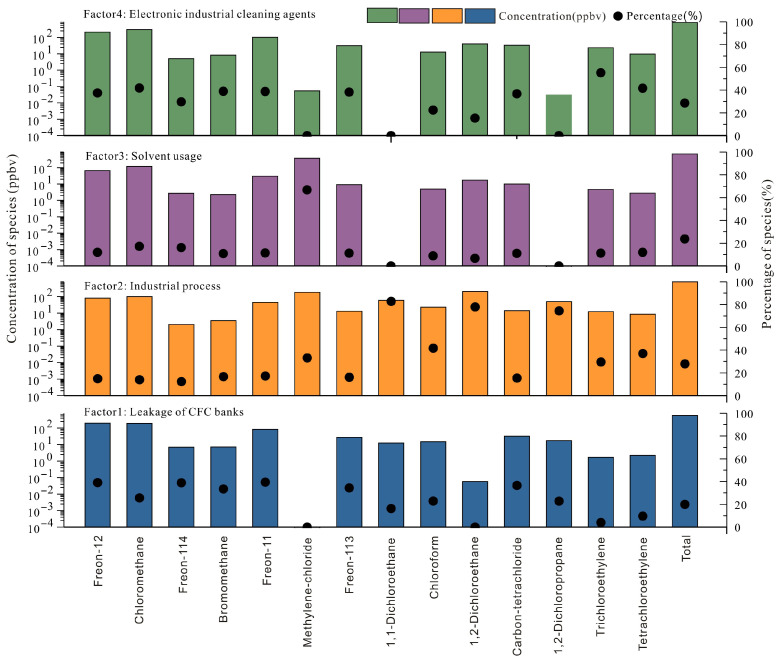
Source profiles of volatile halogenated hydrocarbons (The semi-circle indicates that the contribution proportion of this compound is 0).

Source apportionment of the 14 halocarbons in Mianyang’s summer ambient air was conducted using the PMF 5.0 model. Four factors were resolved, with the resulting source profiles shown in Figure 6. Factor 1 was characterized by high loadings of legacy CFCs, specifically, Freon-114 (42%), carbon tetrachloride (37%), Freon-12 (36%), and Freon-11 (33%). Despite being phased out for approximately two decades, these compounds persist in the atmosphere due to their long atmospheric lifetimes and ongoing release from banks—the reservoirs of CFCs still contained in existing equipment and products [67]. Accordingly, Factor 1 was attributed to leakage from chlorofluorocarbon banks.

Factor 2 was characterized by high contributions from 1,1-dichloroethane (83%), 1,2-dichloroethane (78%), and 1,2-dichloropropane (74%). These compounds are widely employed in industrial processes such as organic synthesis feedstocks, organic solvents, and industrial coatings [17]. Factor 2 was therefore identified as industrial process emissions. Factor 3 was dominated by methylene chloride, which contributed 67% of the factor mass. Owing to its high volatility and solvency, methylene chloride is widely employed as an industrial solvent, as well as a foam blowing agent and chemical feedstock [14,60]. Methyl chloride contributed 19% to this factor and is extensively used in industrial adhesives, sealants, and coatings [53]. Factor 3 was therefore identified as industrial solvent usage. Factor 4 was characterized by high contributions from trichloroethylene (55%) and tetrachloroethylene (41%), both of which are extensively used as cleaning agents in the electronics industry and metal degreasers [40,68]. Factor 4 was identified as emissions from electronics industry cleaning agents. Quantitative source contribution estimates indicated that electronics industry cleaning agents (28.6%) accounted for the largest share of ambient CFCs and VSLSs in Mianyang during summer, followed by industrial process emissions (27.8%), industrial solvent usage (23.7%), and leakage of CFCs banks (19.9%). These results indicate that the phase-out of Montreal Protocol-regulated substances has proven effective in the Sichuan Basin, while the substantial emissions of VSLSs arising from industrial production and solvent usage warrant particular attention.

### 3.4. Health Risk Assessment

**Figure 7:**
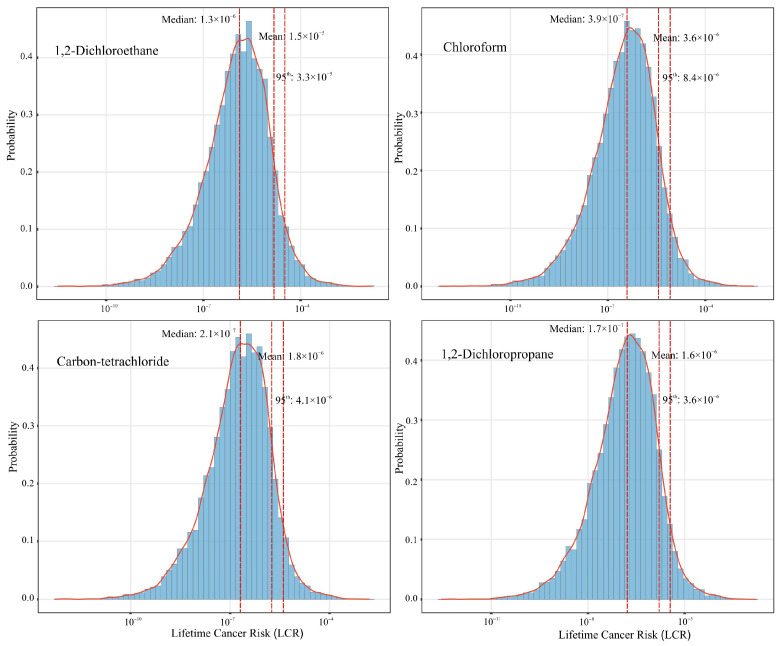
The probability distribution of the estimated inhalation lifetime cancer risk (LCR) for the volatile halocarbons. (The red line represents the lognormal distribution of LCR, and the dashed lines indicate the mean, median, and 95th percentile of the probability distribution).

The lifetime cancer risks of VHHs were assessed using Equations (1) and (2), and the probability distributions of the risks were estimated using Monte Carlo simulation. The lognormal distribution provided the best fit for these compounds. The probability distribution (mean, median and 95th percentile) of the estimated inhalation lifetime cancer risk for these volatile halogenated hydrocarbons are presented in Figure 7.

The threshold of cancer risk for public health protection ranges from one in a million (1.0 × 10^−6^, acceptable risk level) to one in ten thousand (1.0 × 10^−4^, tolerable risk level) [30]. Based on the published IUR parameters, the lifetime cancer risk (LCR) was calculated for eight volatile halogenated hydrocarbons [32]. As the mean values for four of these compounds—trichloroethylene, 1,1-dichloroethane, tetrachloroethylene, and methylene chloride—fell below the acceptable risk threshold of 1 × 10^−6^, indicating negligible cancer risk, they are not depicted in Figure 7. Four volatile halogenated hydrocarbons presented mean cancer risks above the acceptable risk level of 1 × 10^−6^, indicating potential carcinogenic risk. 1,2-Dichloroethane showed the highest mean cancer risks of 1.5 × 10^−5^, followed by chloroform (3.6 × 10^−6^), carbon-tetrachloride (1.8 × 10^−6^), and 1,2-dichloropropane (1.6 × 10^−6^). 1,2-Dichloroethane has also been identified as the compound with the highest carcinogenic risk in some previous studies. The values obtained in this study (1.5 × 10^−5^) were lower than the estimates reported for Zibo City (2.0 × 10^−5^) in Shandong Province and Nanjing City (1.0 × 10^−5^ to 1.0 × 10^−4^) in Jiangsu Province [43,69]. Notably, the carcinogenic risk of 1,2-dichloroethane in this study was 1.4 times the risk value of benzene, a Group 1 human carcinogen, when compared with concurrent studies from our research group [50]. In this study, the average cumulative carcinogenic risk (the sum of the risks from the eight target VHHs) was 2.3 × 10^−5^. Compared with other studies, the cumulative carcinogenic risks were higher than the values reported for Beijing (1.4 × 10^−5^), but lower than those reported for Zibo City (4.9 × 10^−5^) [43,70]. In summary, from the perspective of compound-specific carcinogenic risks, 1,2-dichloroethane warrants greater attention during summer in Mianyang City, as do chloroform, carbon-tetrachloride, and 1,2-dichloropropane, all of which exceeded the safety threshold.

**Figure 8:**
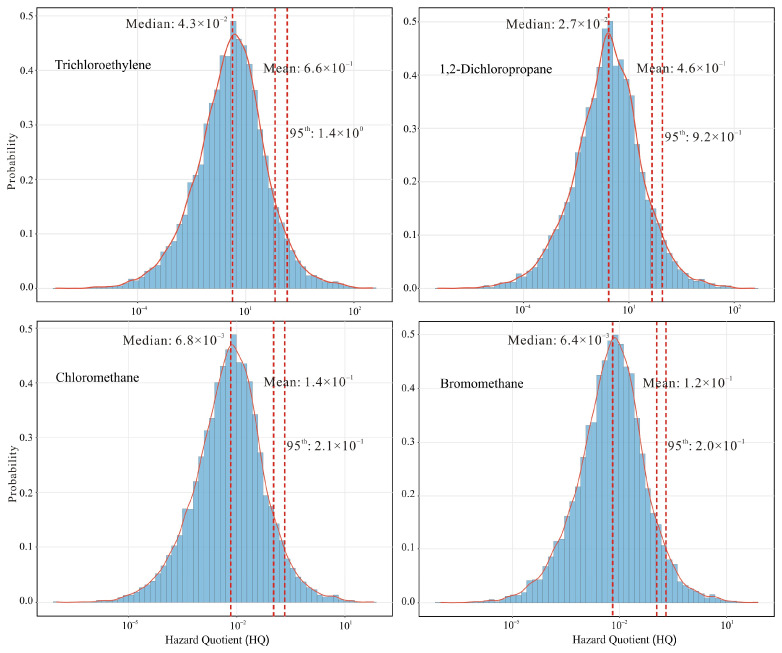
The probability distribution of the estimated inhalation non-carcinogenic risks (Hazard quotient, HQ) for the volatile halocarbons. (The red line represents the lognormal distribution of HQ, and the dashed lines indicate the mean, median, and 95th percentile of the probability distribution).

For 10 VHHs (i.e., trichloroethylene, 1,2-dichloropropane, chloromethane, bromomethane, carbon-tetrachloride, tetrachloroethylene, methylene-chloride, chloroform, 1,2-dichloroethane, and 1,1-dichloroethane) with published RfCs in the IRIS or OEHHA database, non-carcinogenic risk analysis was assessed using Equations (1), (3) and (4). Probability distributions for these compounds were also derived using Monte Carlo simulation. Cumulative non-carcinogenic risk is expressed as the hazard index (HI). An HI value below 1.0 indicates no significant risk of adverse health effects, whereas a value exceeding 1.0 suggests a potential for non-carcinogenic effects [30]. In this study, the HI for the 10 observed species was calculated to be 1.5, indicating a potential non-carcinogenic risk, although all individual compounds yielded mean hazard quotients (HQ) below the non-carcinogenic risk threshold. The four compounds (i.e., trichloroethylene, 1,2-dichloropropane, chloromethane, and bromomethane) with the highest contributions to the HI are identified, and their probability distributions for non-carcinogenic risks are shown in Figure 8. Specifically, trichloroethylene accounted for the largest share of the HI (44.9%), followed by 1,2-dichloropropane (31.2%), chloromethane (9.3%), and bromomethane (8.4%). At the upper-bound 95th percentile for non-cancer risk estimates, the HQ for trichloroethylene exceeded the safety threshold; moreover, the HI for the 10 VHHs was 2.9 times the acceptable limit.

**Figure 9:**
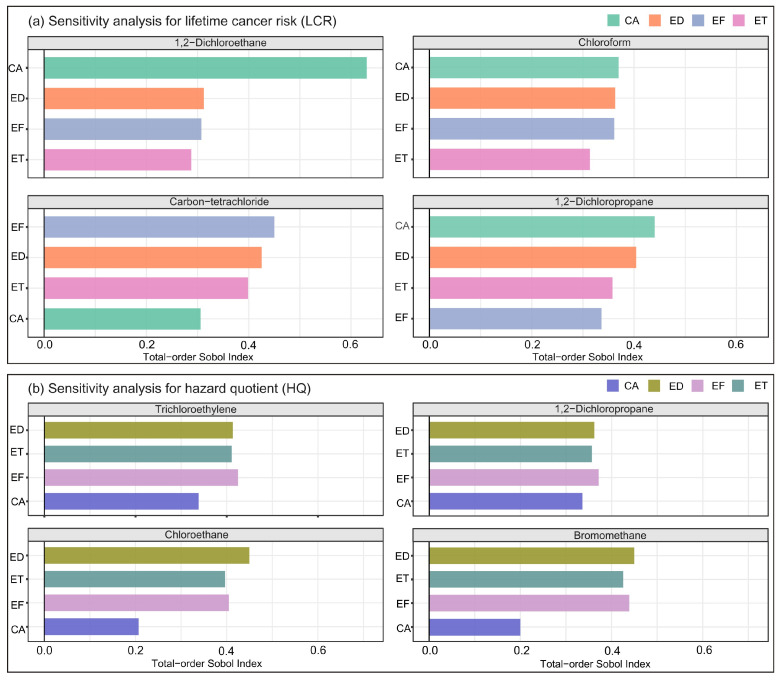
(**a**) Sensitivity analysis for the four compounds exhibiting the highest carcinogenic risks (LCR) and (**b**) for those with the highest non-carcinogenic risks (HQ).

The contribution of each input parameter to the carcinogenic and non-carcinogenic risks (LCR and HQ) was further assessed using a sensitivity analysis based on the Sobol indices method. Figure 9 presents the sensitivity analysis results for four compounds exhibiting the highest carcinogenic and non-carcinogenic risks, respectively. For 1,2-dichloroethane, chloroform and 1,2-dichloropropane, the CA factor showed the highest total-order Sobol index, indicating that CA is the dominant contributor to the variance in their carcinogenic risk estimates (Figure 9a). For carbon tetrachloride, the EF factor exhibited the highest total-order Sobol index, with the indices for other time-related parameters also being elevated. This may be attributed to the IUR for carbon tetrachloride being an order of magnitude lower than those for the other three compounds. Sensitivity analysis for non-carcinogenic risks revealed consistently high contributions from EF, ED, and ET (Figure 9b). Consistent with previous studies, these findings underscore the pivotal role of pollutant concentrations and time-related parameters in determining inhalation exposure risk [35,36,39].

## 4. Conclusions

In this study, 14 ambient volatile halocarbons were analyzed at a site at Southwest University of Science and Technology in Mianyang City during a prolonged period of high temperatures and no precipitation in the summer of 2019. The results showed that the six Montreal Protocol–controlled CFCs maintained relatively stable mixing ratios, ranging from 987 to 1129 pptv, while the eight unregulated VSLSs varied from 937 to 5066 pptv—collectively comprising a substantial fraction (64.5% on average) of the total 14 volatile halocarbons. Among the CFCs, Freon-12, Freon-11, and carbon tetrachloride were the predominant species, while chloromethane, methyl chloride, and 1,2-dichloroethane were the most abundant among the VSLSs. These six species accounted for 86% of the total volatile halocarbons during the observation period.

A striking disparity was observed between regulated and unregulated species when comparing their mixing ratios to global background levels. While phased-out CFCs exhibited only modest enhancements (5–16%), the unregulated VSLSs showed pronounced increases ranging from 59% to an exceptional 2401%—with trichloroethylene, methylene chloride, and tetrachloroethylene peaking at 2401%, 1050%, and 704%, respectively. Among the phased-out CFCs, Freon-114 exhibited the smallest deviation from background levels (5%, 1 pptv), followed by Freon-12 (12.4%, 62 pptv) and Freon-11 (16.2%, 37 pptv); bromomethane, in contrast, displayed the highest enhancement (223%, 15 pptv). This contrast underscores the effectiveness of Montreal Protocol controls in curbing CFCs emissions, while highlighting the growing atmospheric burden of unregulated VSLSs as observed in Mianyang.

Health risk assessment revealed that four volatile halocarbons, 1,2-dichloroethane, chloroform, carbon tetrachloride, and 1,2-dichloropropane, posed potential carcinogenic risks to the local population. 1,2-dichloroethane exceeded the safety threshold for cancer risk by a factor of 15, and the average cumulative carcinogenic risk surpasses the acceptable level by a factor of 23. Meanwhile, the cumulative exposure of all VHHs posed potentially significant non-carcinogenic risks, with trichloroethylene and 1,2-dichloropropane being the predominant contributors (76.1%).

PMF model analysis revealed the main sources of volatile halocarbons in Mianyang during summer. Electronic industrial solvents (28.6%) and industrial emissions (27.8%) were the dominant sources, followed by industrial solvents and raw materials (23.7%) and leakage from CFCs banks (19.9%). Among the species of concern, 1,2-dichloroethane, chloroform, and 1,2-dichloropropane are key contributors to the regional carcinogenic risk and were traced largely to industrial process emissions. Meanwhile, trichloroethylene, the principal driver of non-carcinogenic risk, originated mainly from cleaning agents used in electronics manufacturing and metal degreasing.

The findings indicate that local authorities have effectively curtailed the emissions of most substances phased out under the Montreal Protocol; while also highlighting the urgent need for strengthened regulation of CFCs substitutes and short-lived halocarbons is evident in this region. Several limitations should be outlined alongside a brief discussion of future work. The sampling campaign was constrained by a relatively short duration and a limited number of samples. Although this dataset is sufficient to characterize a typical summer pollution episode of halogenated hydrocarbons in a medium-sized urban area of the Sichuan Basin, it offers only a restricted basis for assessing seasonal or interannual variability under differing emission scenarios. The toxicity data for VHHs used in this study were sourced mainly from the USEPA. Such reliance on a single external database may introduce a degree of uncertainty when assessing the health implications for the Chinese population. The source attributions for halocarbon loadings presented in this study remain preliminary. Subsequent investigations could incorporate near-source sampling campaigns specifically designed to validate the chemical signatures of individual emitters. Additionally, the deployment of multi-site, long-term observational networks across the Sichuan Basin would enable a more refined analysis of spatiotemporal variability and the emission dynamics governing both regulated and unregulated halocarbon species.

## Figures and Tables

**Table 1:** Mixing ratios (pptv), atmospheric lifetimes (year), ozone depletion potentials (ODP), global warming potentials (GWP_100_) of CFCs and VSLSs in this study and comparison with other studies.

Species	This Study (pptv ± SD)	Global Background ^abc^	The Yellow Rivere Stuary [21]	Hangzhou [26]	Hongkong [44]	Lifetime [7]	ODP [7]	GWP_100_ [7]
Freon-12 ^a^	563 ± 20	501 ± 1.4	529 + 14	/	568 + 22	102	0.75	12,500
Freon-114 ^b^	17 ± 4	16 ± 0.5	17 + 0.6	20	19 + 1.7	189	0.53	9450
Bromomethane ^b^	21 ± 1	6.6 ± 0.3	25 + 23	12	15 + 19	0.80	0.57	2
Freon-11 ^a^	264 ± 15	227 ± 0.5	274 + 26	229	244 + 26	52	1	6410
Freon-113 ^a^	81 ± 3	71 ± 0.4	83 + 5	/	76 + 24	93	0.82	6530
Carbon-tetrachloride ^a^	92 ± 9	78 ± 0.4	117 + 37	79	85 + 12	32	0.87	2150
Chloromethane ^b^	785 ± 261	493 ± 20	1879 + 1336	912	828 + 160	0.90	0.02	6
Methylene chloride ^b^	563 ± 505	49 ± 3.5	723 + 467	2207	267 + 355	0.49	/	11
1,1-Dichloroethane ^a^	73 ± 47	/	/	58	/	0.37	/	4
Chloroform ^b^	64 ± 39	9.8 ± 1.0	378 + 310	129	44 + 55	0.50	/	20
1,2-Dichloroethane ^a^	265 ± 279	/	586 ± 511	596	/	0.23	/	1
1,2-Dichloropropane ^a^	67 ± 37	/	/	306	/	0.07	/	/
Trichloroethylene ^b^	42 ± 10	1.7 ± 1.1	97 + 360	50	25 + 58	0.02 ^c^	/	<<1
Tetrachloroethylene ^c^	27 ± 15	3.3 ± 1.4	27 + 20	57	24 + 31	0.30	/	6

^a^ Data source: Mauna Loa (19.539° N, 155.578° W) of the NOAA Global Monitoring Laboratory (GML); https://gml.noaa.gov/data/data.php?category=Halocompounds (accessed on 10 March 2026); ^b^ Data source: Jungfraujoch (46.548° N, 7.990° E) of the NASA Advanced Global Atmospheric Gases Experiment (AGAGE) network; https://www-air.larc.nasa.gov/missions/agage/data (accessed on 10 March 2026); ^c^ Data source: Monte Cimone (44.193° N, 10.701° E) of the NASA Advanced Global Atmospheric Gases Experiment (AGAGE) network; https://www-air.larc.nasa.gov/missions/agage/data (accessed on 10 March 2026).

## Data Availability

The original contributions presented in this study are included in the article. Further inquiries can be directed to the corresponding authors.

## Abbreviations

The following abbreviations are used in this manuscript:

VHHs	Volatile halogenated hydrocarbons
ODSs	Ozone-depleting substances
CFCs	Chlorofluorocarbons
VSLSs	Very short-lived substances
PMF	Positive Matrix Factorization
LCR	Lifetime cancer risk
HQ	Hazard quotients
HI	Hazard index
